# Supplementary material for: Conditional chemoconnectomics (cCCTomics) as a strategy for efficient and conditional targeting of chemical transmission
Source: eLife. 2024 Apr 30;12:RP91927. doi: 10.7554/eLife.91927 (PMC11060718; doi:10.7554/eLife.91927)
Supplement: Figure 3—source data 3. [file elife-91927-fig3-data3.zip › Figure 3 source data3 part 2.zip!/Figure 3-Source data 3-part2/1025-13-CRISPResso_Report_ack040l3.zip!/CRISPResso_on_1025_16/9.Alleles_frequency_table_around_sgRNA_agcacaaacttggcggtgac.pdf]

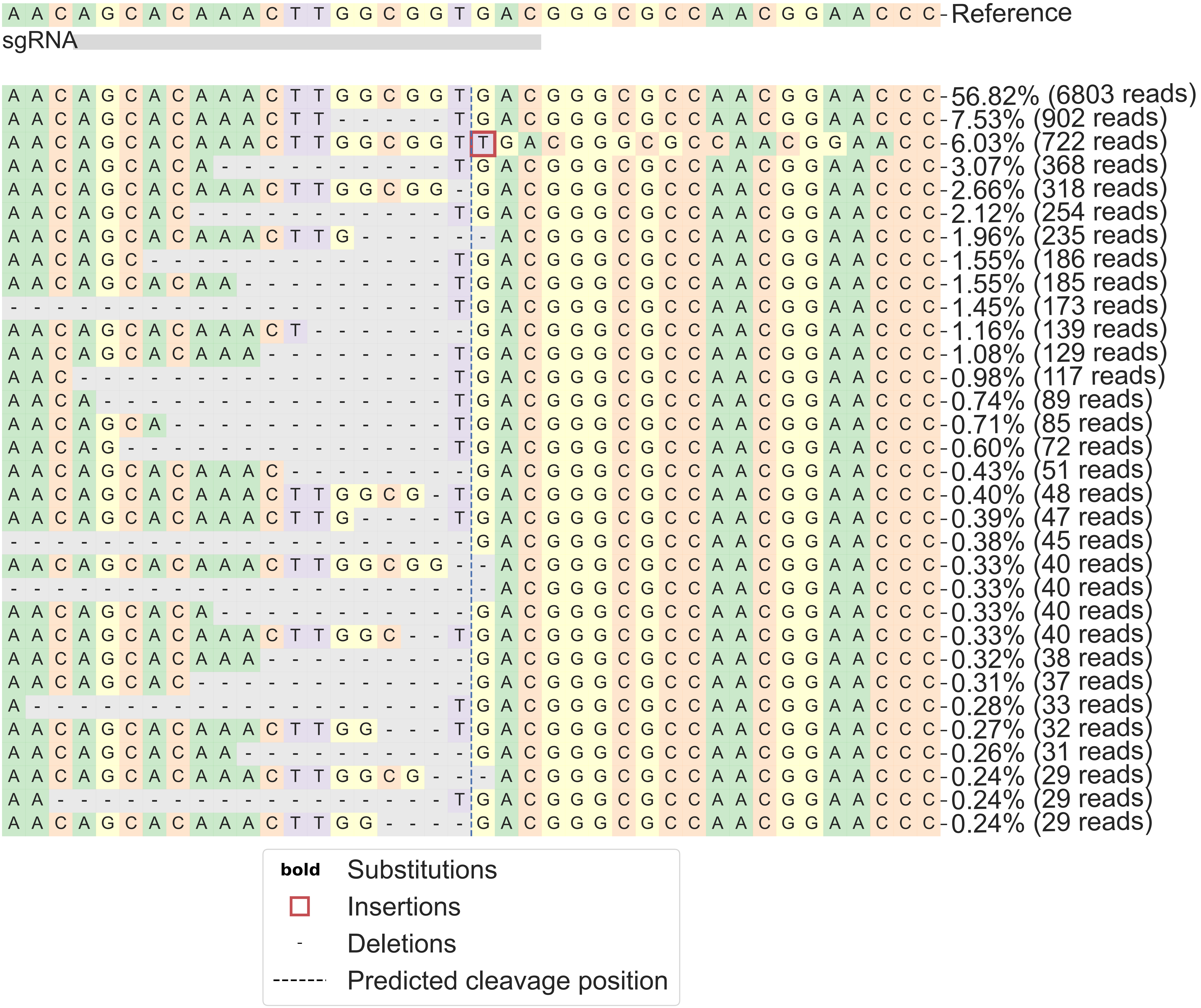
AACAGCACAAACTTGGCGGTGACGGGCGCCAACGGAACCC Reference
sgRNA
AACAGCACAAACTTGGCGGTGACGGGCGCCAACGGAACCC 56.82% (6803 reads)
AACAGCACAAACTT-----TGACGGGCGCCAACGGAACCC 7.53% (902 reads)
AACAGCACAAACTTGGCGGTTGACGGGCGCCAACGGAACC 6.03% (722 reads)
AACAGCACA----------TGACGGGCGCCAACGGAACCC 3.07% (368 reads)
AACAGCACAAACTTGGCGG-GACGGGCGCCAACGGAACCC 2.66% (318 reads)
AACAGCAC-----------TGACGGGCGCCAACGGAACCC 2.12% (254 reads)
AACAGCACAAACTTG------ACGGGCGCCAACGGAACCC 1.96% (235 reads)
AACAGC-------------TGACGGGCGCCAACGGAACCC 1.55% (186 reads)
AACAGCACAA---------TGACGGGCGCCAACGGAACCC 1.55% (185 reads)
-------------------TGACGGGCGCCAACGGAACCC 1.45% (173 reads)
AACAGCACAAACT-------GACGGGCGCCAACGGAACCC 1.16% (139 reads)
AACAGCACAAA--------TGACGGGCGCCAACGGAACCC 1.08% (129 reads)
AAC----------------TGACGGGCGCCAACGGAACCC 0.98% (117 reads)
AACA---------------TGACGGGCGCCAACGGAACCC 0.74% (89 reads)
AACAGCA------------TGACGGGCGCCAACGGAACCC 0.71% (85 reads)
AACAG--------------TGACGGGCGCCAACGGAACCC 0.60% (72 reads)
AACAGCACAAAC--------GACGGGCGCCAACGGAACCC 0.43% (51 reads)
AACAGCACAAACTTGGCG-TGACGGGCGCCAACGGAACCC 0.40% (48 reads)
AACAGCACAAACTTG----TGACGGGCGCCAACGGAACCC 0.39% (47 reads)
--------------------GACGGGCGCCAACGGAACCC 0.38% (45 reads)
AACAGCACAAACTTGGCGG--ACGGGCGCCAACGGAACCC 0.33% (40 reads)
---------------------ACGGGCGCCAACGGAACCC 0.33% (40 reads)
AACAGCACA-----------GACGGGCGCCAACGGAACCC 0.33% (40 reads)
AACAGCACAAACTTGGC--TGACGGGCGCCAACGGAACCC 0.33% (40 reads)
AACAGCACAAA---------GACGGGCGCCAACGGAACCC 0.32% (38 reads)
AACAGCAC------------GACGGGCGCCAACGGAACCC 0.31% (37 reads)
A------------------TGACGGGCGCCAACGGAACCC 0.28% (33 reads)
AACAGCACAAACTTGG---TGACGGGCGCCAACGGAACCC 0.27% (32 reads)
AACAGCACAA----------GACGGGCGCCAACGGAACCC 0.26% (31 reads)
AACAGCACAAACTTGGCG---ACGGGCGCCAACGGAACCC 0.24% (29 reads)
AA-----------------TGACGGGCGCCAACGGAACCC 0.24% (29 reads)
AACAGCACAAACTTGG----GACGGGCGCCAACGGAACCC 0.24% (29 reads)
bold Substitutions
Insertions
Deletions
Predicted cleavage position
